# Critical Role of Monooxygenase in Biodegradation of 2,4,6-Trinitrotoluene by *Buttiauxella* sp. S19-1

**DOI:** 10.3390/molecules28041969

**Published:** 2023-02-19

**Authors:** Miao Xu, Lei He, Ping Sun, Ming Wu, Xiyan Cui, Dong Liu, Amma G. Adomako-Bonsu, Min Geng, Guangming Xiong, Liquan Guo, Edmund Maser

**Affiliations:** 1 College of Life Science, Jilin Agricultural University, Changchun 130018, China; 2 Grain School, Jilin Busyness and Technology College, Changchun 130507, China; 3 Institute of Toxicology and Pharmacology, University Medical School Schleswig-Holstein, 24105 Kiel, Germany; 4 School of Food and Biotechnology, Changchun Polytechnic, Changchun 130033, China

**Keywords:** *Buttiauxella* sp. S19-1, 2,4,6-trinitrotoluene (TNT), monooxygenase (MO), bioremediation, genetic manipulation

## Abstract

2,4,6-Trinitrotoluene (TNT) is an aromatic pollutant that is difficult to be degraded in the natural environment. The screening of efficient degrading bacteria for bioremediation of TNT has received much attention from scholars. In this paper, transcriptome analysis of the efficient degrading bacterium *Buttiauxella* sp. S19-1 revealed that the monooxygenase gene (*BuMO*) was significantly up-regulated during TNT degradation. S-Δ*MO* (absence of *BuMO* gene in S19-1 mutant) degraded TNT 1.66-fold less efficiently than strain S19-1 (from 71.2% to 42.9%), and E-*MO* mutant (*Escherichia coli BuMO*-expressing strain) increased the efficiency of TNT degradation 1.33-fold (from 52.1% to 69.5%) for 9 h at 180 rpm at 27 °C in LB medium with 1.4 µg·mL^−1^ TNT. We predicted the structure of BuMO and purified recombinant BuMO (rBuMO). Its specific activity was 1.81 µmol·min^−1^·mg^−1^ protein at pH 7.5 and 35 °C. The results of gas chromatography mass spectrometry (GC–MS) analysis indicated that 4-amino-2,6-dinitrotoluene (ADNT) is a metabolite of TNT biodegradation. We speculate that MO is involved in catalysis in the bacterial degradation pathway of TNT in TNT-polluted environment.

## 1. Introduction

The potent explosive 2,4,6-trinitrotoluene (TNT) has been used extensively in the military since 1863 [1,2]. With the exception of mine barriers, failed detonations, downed military aircrafts, and sunken ships loaded with munitions, huge amounts of various unused war materials were thrown into the oceans after the First and Second World Wars [3]. For example, in Northern Germany and the Baltic Sea region alone, there are about two million metric tons of intense explosives (including TNT) [4]. Gradual corrosion of these dumped munitions over the years has resulted in leakage of a cocktail of explosive compounds, including TNT in the oceans [3,5], with the detection of significant levels of TNT and its metabolites in sea water [6,7]. Unlike other nitroaromatic compounds, TNT has high water solubility (130 mg·L^−1^) [8]. Its recalcitrance, due to the presence of symmetrical electron-withdrawing nitro substituents groups [9], contributes significantly towards its environmental persistence. Therefore, the proliferation of TNT in oceans has become a worldwide problem.

Furthermore, leaked munition compounds (such as TNT) in the oceans are absorbed by aquatic organisms [4,10,11,12,13], posing a significant threat to the marine ecosystem and the health of seafood consumers. TNT has been considered as toxic, mutagenic, and carcinogenic to humans [14,15]. Therefore, the removal of TNT from the environment is crucial for the sustenance and safety of the ecosystem and humans.

Remediation of TNT from the environment has been a widespread concern for decades. Physical methods such as adsorption [16], extraction [17,18], incineration [19], and chemical methods such as ozone and the combined ozone method [20], Fenton process and Fenton-like process [21], and the wet air oxidation process (WAO) [22] have been explored. Compared to physical and chemical degradation methods, bioremediation is regarded as the most economical and efficient way to completely mineralise TNT and its metabolites [23,24]. Since TNT is readily reducible in natural media to produce 2-amino-4,6-dinitrotoluene, 4-amino-2,6-dinitrotoluene (ADNT), and their isomers, much research have focused on investigating the possible oxidative transformation of these isomers. Oxygenases are particularly important in this process [25].

Oxygenases are capable of metabolising organic compounds, improving their reactivity, water solubility, and cleaving organic rings by incorporating molecular oxygen [26]. These enzymes can be divided into dioxygenases (which catalyse the binding of diatomic oxygen from the oxygen molecule to the substrate) and monooxygenases (MOs—which incorporate only one atomic oxygen in the presence of a hydrogen donor, such as NADPH) [27]. At present, the most studied types of MOs include flavin MO, p450 MO, polysaccharide MO, and methane MO; these MOs participated in the biodegradation of benzene, polycyclic aromatic hydrocarbons (PAHs), persistent organic pollutants, and other macrocyclic substances. For example, *Pseudomonas* sp. BCNU 106 degrades benzene compounds via toluene dioxygenase and xylene MO [28]. Detoxifying CYP5035S7 MO of the White-Rot Fungus *Polyporus arcularius* hydroxylates (N)PAHs and participates in the detoxification of xenobiotics by fungus [29]. Hexachlorobenzene MO HcbA1A3 was reported as an aerobic HCB dechlorinase [30]. Therefore, elucidation of the potential role of MOs in TNT biodegradation could be a major boost to bioremediation efforts, considering its involvement in xenobiotic metabolism (particularly macrocyclic compounds).

In previous TNT biodegradation studies, recombinant nitrobenzene oxygenase from *Comamonas* sp. JS765 oxidized ADNT and formed 3-amino-6-methyl-5-nitrocatechol [31]. A combination of terminal oxygenase-2,4-dinitrotoluene oxygenase and nitrobenzene oxygenase leads to simultaneous oxidation of the corresponding TNT derivatives to form benzyl alcohol and catechol [32]. However, the participation of MO in the biodegradation of TNT has not been reported; MO maybe play a critical role in TNT degradation.

A recent assessment of the macrocyclic-degrading strains such as *Pseudomonas putida*, *Pseudomonas* sp., *Pseudomonas stutzeri*, *Rhodococcus* sp., and *Buttiauxella* sp. revealed that *Buttiauxella* sp. S19-1 was found to be the most efficient TNT-degrading bacterium [33]. Additionally, prokaryotic transcriptome analysis showed significant up-regulation of MO following the exposure of strain S19-1 to TNT. Herein, to better illuminate the TNT biodegradation pathway in strain S19-1, the role of MO in the TNT biodegradation pathway will be elucidated through gene functional analysis and gene manipulation, as well as intermediate metabolite detection.

## 2. Results and Discussion

TNT content remaining in the LB medium was analysed via high-performance liquid chromatography (HPLC). TNT levels in strain S19-1 culture after 9-h incubation decreased by 87.5% (*p* < 0.05). Correspondingly, the content of TNT in culture medium without strain S19-1 almost did not decrease. The results indicated TNT was taken up and degraded by strain S19-1. This finding is comparable to previous results and confirm the robustness of strain S19-1 as a model in TNT biodegradation studies [33].

### 2.1. Transcriptomic Analysis of Strain S19-1

#### 2.1.1. Sequencing Data Filtering

To ensure the quality of the analysed information, some invalid data (such as high N content of unknown bases and reads with low quality) were excluded. The quality of the filtered reads is presented in Table S1.

#### 2.1.2. Screening of Differentially Expressed Genes (DEGs) during TNT Degradation

**Figure 1:**
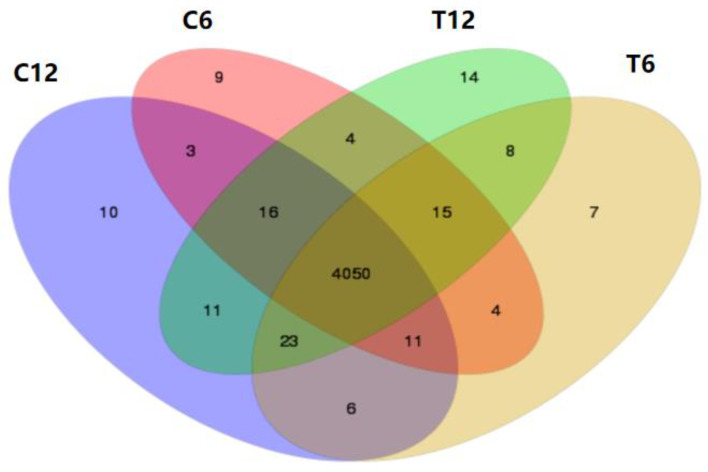
Venn diagram of shared genes between samples.

Venn analysis was performed on the transcriptome results from the four sample groups (C6, C12; and T6, T12). These results are shown in Figure 1. Overall, there were 4050 overlapping genes between the control groups (C6, C12) and the experimental groups (T6, T12). Nine genes were exclusive to C6, whereas 10 genes were only expressed in C12. In T6 samples only, seven genes were exclusive; 14 genes were expressed in the T12 group.

**Figure 2:**
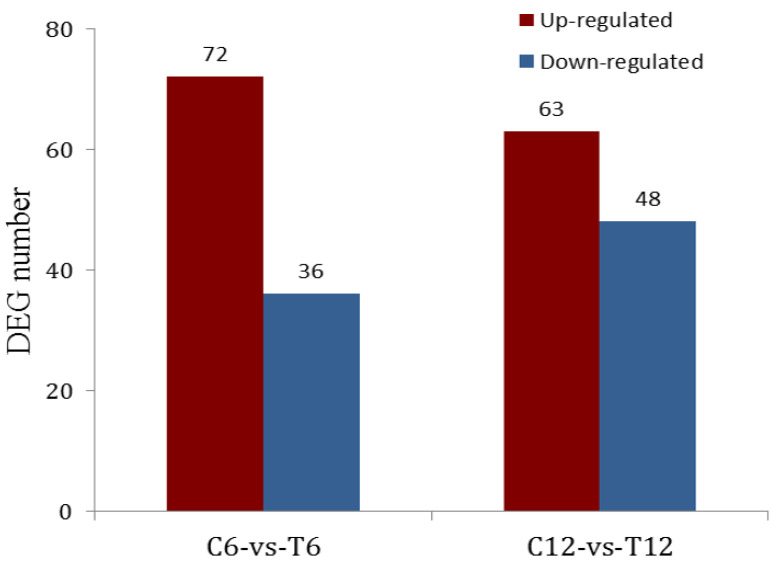
Statistical analysis of DEGs.

Figure 2 shows the analysis of DEGs with 108 DEGs detected (*p* < 0.01) after 6-h incubation with TNT, 72 of which were up-regulated and 36 genes down-regulated. On the other hand, 111 DEGs were detected (*p* < 0.01) after 12 h incubation with TNT-63 and 48 genes were up- and down-regulated, respectively.

The up-regulated genes included antibiotic biosynthesis MO, 1,2-dioxygenase, aldehyde dehydrogenase, oxidoreductase, phosphotransacetylase, alkaline phosphatase, lysine decarboxylase, and other enzymes that could participate in TNT degradation. For quantitative polymerase chain reactions (qPCR) identification of the transcriptome data, the top 10 DEGs in the above two groups (6 h/12 h) with significant up-regulation were selected (Table S2).

#### 2.1.3. The Gene Ontology (GO) Term Enrichment Analysis of DEGs

In view of the gene function classification GO database, the overall analysis and evaluation of the DEGs related to TNT degradation in strain S19-1 was performed (*p* < 0.05), mainly involving the enrichment analysis of biological processes, molecular function, and cellular components (the first 30 items and their abundance values are displayed in Figure S1A).

A total of 364 genes, with different functions, were enriched in strain S19-1 after 6 h incubation with TNT. As show in Figure S1B, the GO annotation enrichment analysis showed 121 genes corresponding to biological processes; these genes are involved in cellular processes (34 genes, accounting for 58.6% of the gene cluster frequency), metabolic processes (33 genes, accounting for 59.6% of the gene cluster frequency), and localization (26 genes, accounting for 49.2% of the gene cluster frequency). These results indicate that the physiological and metabolic processes of strain S19-1 were affected, to a certain extent, after exposure to TNT. For example, genes D8682_RS09495 (lysine decarboxylase) and D8682_RS03965 (pyridoxine kinase), among others (which are involved in the metabolic processes of strain S19-1), were enriched after TNT exposure. Genes corresponding to cellular component (142 genes) and molecular function (101 genes) were also enriched after TNT exposure.

In the cellular component, enrichment was recorded in the cell (36 genes, accounting for 69.2% of the gene cluster frequency), cell parts (36 genes, accounting for 69.2% of the gene cluster frequency), the cell membrane (33 genes, accounting for 63.2% of the gene cluster frequency), and cell membrane parts (30 genes, accounting for 57.7% of the gene cluster frequency). Membrane-related genes up-regulated after TNT exposure included gene D8682_RS01950 (iron (III) transport system permease protein), gene D8682_RS04560 (integral component of membrane), and gene D8682_RS09020 (basic amino acid-specific outer membrane pore). These findings indicate that genes peculiar to membrane transport (belonging to cellular components) may have been mobilized to participate in the recognition and transmembrane transport of TNT during exposure and, ultimately, degradation (Figure S1B).

For molecular function, enrichment was mainly observed in catalytic activity (43 genes, accounting for 62.3% of the gene cluster frequency), binding (32 genes, accounting for 46.4% of the gene cluster frequency), and transporter activity (19 genes, accounting for 27.5% of the gene cluster frequency). Several genes related to TNT degradation and metabolism, such as gene D8682_RS06515 (phosphotransferase (PTS) system, glucose-specific IIC component), gene D8682_RS07115 (multidrug efflux pump), and gene D8682_RS13195 (PTS system, ascorbate-specific IIA component), were enriched. The up-/down-regulation of gene expression among the main enriched functions was further analysed (Figure S1B).

With regard to biological process and molecular function, genes of the main enrichment pathways were predominantly up-regulated. This indicates that the up-regulated genes in strain S19-1 were involved in the identification, transport, and degradation of TNT, independent of TNT toxicity. Enrichment in the cellular components was mainly in the cell and cell parts, and the number of up- and down-regulated genes was similar. Therefore, one could infer that incubation of strain S19-1 with TNT resulted in certain physiological responses to TNT-induced stress (which could be modulated via these genes).

GO annotation enrichment analysis after TNT degradation in strain S19-1 (12 h) showed biological processes (91 genes), cellular components (100 genes), and molecular function (80 genes). As shown in Figure S2A, enrichment in the genes of the biological processes corresponded to cellular (30 genes) and metabolic processes (28 genes), similar to 6 h of TNT exposure. However, the number of up-regulated genes decreased, perhaps due to the decrease in TNT concentration in the bacterial culture.

Enrichment in the cellular component resulted in up-regulation of genes in membrane (25 genes), membrane parts (24 genes), cell (23 genes), and cell parts (22 genes). The enrichment results of membrane-related genes also indicated that some genes were involved in recognition of TNT during degradation in strain S19-1 (12 h exposure). These genes, such as gene D8682_RS09495 (lysine decarboxylase) and gene (aspartate 1-decarboxylase) may have been up-regulated to assist with transmembrane transport and, ultimately, complete degradation of TNT. Compared to the 6 h group, enrichment in molecular function, particularly in catalytic activity (37 genes) and binding (28 genes), was lower. This might be due to the promotion of TNT degradation with longer treatment time, while the expressions of some other genes were close to normal levels.

As shown in Figure S2B, the overall gene expression trend in biological processes and molecular function for the 12 h group (compared to the 6 h group) changed; the number of up-regulated genes was less than that of down-regulated genes. Cellular component parts were significantly enriched in the membrane, membrane parts, cell, and cell parts, and the overall gene expression trend was similar to that of the 6 h group, indicating that the stability of bacterial cell physiological structure was not affected by time.

#### 2.1.4. Kyoto Encyclopaedia of Genes and Genomes (KEGG) Enrichment Analysis of Differential Genes

After 6-h incubation with TNT, the top-ranked KEGG-enriched pathway of strain S19-1 showed significant changes in genes that could be involved in the recognition, transport, and metabolism of TNT. As shown in Figure S3A, enrichment was observed after 6-h incubation with TNT (in comparison to control samples), including cellular community-prokaryotes (six genes), cell motility (one gene), membrane transport (13 genes), signal transduction (two genes), translation (two genes), replication and repair (one gene), global and overview maps (23 genes), carbohydrate metabolism (18 genes), metabolism of cofactors (eight genes), amino acid metabolism (seven genes), nucleotide (two genes), xenobiotics biodegradation and metabolism (two genes), biosynthesis of other secondary metabolites (one gene), and metabolism of other amino acids (one gene). In the carbohydrate metabolism category, enriched genes corresponded to hydrocarbon metabolism and biodegradation of heterologous biomass containing benzene ring structures. The distribution of DEGs in each enriched pathway was further analysed (Figure S3B). Up-regulated genes were the main pathways that may play a leading role in TNT degradation, including novobiocin biosynthesis, taurine, hypotaurine metabolism, and butanoate metabolism, etc. Altered gene expression was also observed in fructose and mannose metabolism, phosphotransferase system, alanine, aspartate, and glutamate metabolism, etc. Overall, a large number of genes participated in the process of TNT degradation.

The overall distribution characteristics of the KEGG enrichment results from the 12 h exposure groups (shown in Figure S4A) were similar to those of the 6 h exposure groups, indicating that physiological pathways were not affected by time. The D8682_RS11975 (MO) gene was up-regulated in strain S19-1 following 6 h and 12 h incubation with TNT and could therefore be a participant in TNT degradation. Furthermore, the results show that only genes of ascorbate and aldarate metabolism, taurine and hypotaurine metabolism, biofilm formation, lysine degradation, thiamine metabolism, and pyruvate metabolism were up-regulated, suggesting that the above pathways were either participants in the process of TNT degradation or related to the adaptability of this strain to TNT (Figure S4B).

In the 12 h exposure group, the total count of down-regulated genes in metabolic processes, the biosynthesis of secondary metabolites, the biosynthesis of antibiotics, purine metabolism, and microbial metabolism was higher than up-regulated genes, showing an opposite trend to that of the 6 h exposure group. Meanwhile, purine metabolism, one carbon pool by folate, aminobenzoate degradation, fluorobenzoate degradation, pherylalanine metabolism, toluene degradation, benzoate degradation, xylene degradation, pathway enrichment genes for the degradation of aromatic compounds, tryptophan metabolism, prodigiosin biosynthesis, and other pathways were predominantly down-regulated. Thus, prolongation of the exposure time (12 h incubation with TNT) could inhibit most of the metabolic pathways.

#### 2.1.5. Spatiotemporal Sequence Analysis

Spatiotemporal sequence analysis showed similar expression patterns in both 6 and 12 h exposure cultures of strain S19-1 (Figure S5). These genes could be clustered based on their exposure times. Genes with the same expression pattern could also be clustered together to facilitate the visualization of the effect of different exposure times on gene expression following TNT exposure.

As shown in Figure S5A, the expression pattern of some gene sequences of changed at two different time points (6 h and 12 h). This may be related to the metabolism and utilization of TNT by strain S19-1. For D8682_RS11975 (monooxygenase), gap (glyceraldehyde 3-phosphate dehydrogenase), leuO (LysR family transcriptional regulator), D8682_RS01910 (LuxR family transcriptional regulator), wcaD (putative colanic acid polymerase), D8682_RS02405 (ethanolamine utilization protein), and other genes included in the gene cluster, the expression pattern in wild-type strain S19-1 changed at 6 h and 12 h, after exposure to TNT. After exposure to TNT for 6 h, the expression level decreased significantly compared to the control groups. After exposure to TNT for 12 h, the expression level increased significantly compared to the control groups.

However, spatio-temporal analysis showed that expression changes between control groups and TNT groups were different at 6 h and 12 h. The expression of D8682_RS04155 (major type 1 subunit fimbrin), bioC (malonyl-CoA O-methyltransferase), D8682_RS21965 (sodium/bile acid cotransporter), uca (urea carboxylase), D8682_RS03180 (threonine-protein kinase), urtB (urea transport system permease protein), and tehA (tellurite resistance protein) i increased significantly after exposure to TNT for 12 h (Figure S5B).

#### 2.1.6. Transcriptomic Analysis of BuMO

Transcriptomic analysis showed that editorial genes for key enzymes such as MO were up-regulated upon TNT exposure, and these results are consistent with the findings of Bolt et al. in which reductase and dehydrogenase were considered the key enzymes of the TNT biodegradation pathway [14]. Furthermore, the expression of the *BuMO* gene was significantly increased at 12-h exposure of TNT compared to the control group, with a logarithmic fold change of 2.52548 (Table S3). Hence, subsequent investigations were focused on the functional effects of BuMO in TNT biodegradation.

### 2.2. Bioinformatics Analysis of BuMO

**Figure 3:**
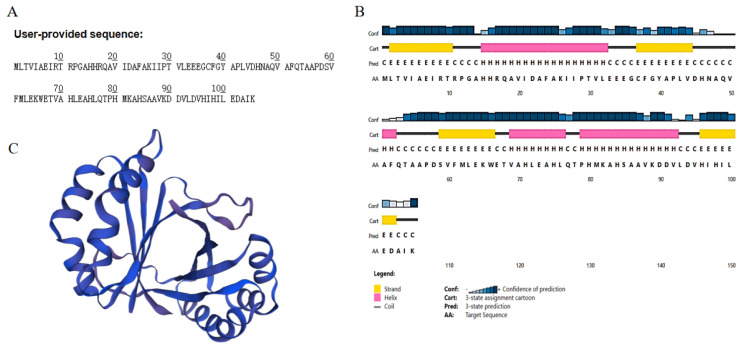
Bioinformatics analysis of BuMO. (**A**)—The primary structure. (**B**)—The secondary structure. (**C**)—The tertiary structure.

The primary structure and physicochemical properties of the protein were analysed (Figure 3A). The results showed that the protein is composed of 105 amino acids with a molecular weight of 11683.36. The chemical formula of the protein is C_523_H_819_N_143_O_150_S_4_. The total atomic number is 1639. The theoretical isoelectric point is 5.61. The half-life of this protein is about 30 h (mammalian erythrocytes, in vitro); >20 h (yeast, in vivo); >10 h (*E. coli*, in vitro). The instability index of the protein was 38.06, indicating that the protein was stable. The fat index of the protein was 102.29. The total average hydrophilicity (GRAVY) of the protein was 0.072.

The prediction of the secondary and tertiary structures of MO showed that the protein contains four α-helices, four β-folds, eight random curls, and the tertiary structure of MO (Figure 3B,C).

### 2.3. Biodegradation of TNT by Mutants

To further clarify the catalytic effect of MO, we determined the specific activity of MO for 4-hydroxybenzaldehyde, which was 1.81 µmol·min^−1^·mg^−1^ protein at pH 7.5 and 35 °C.

**Figure 4:**
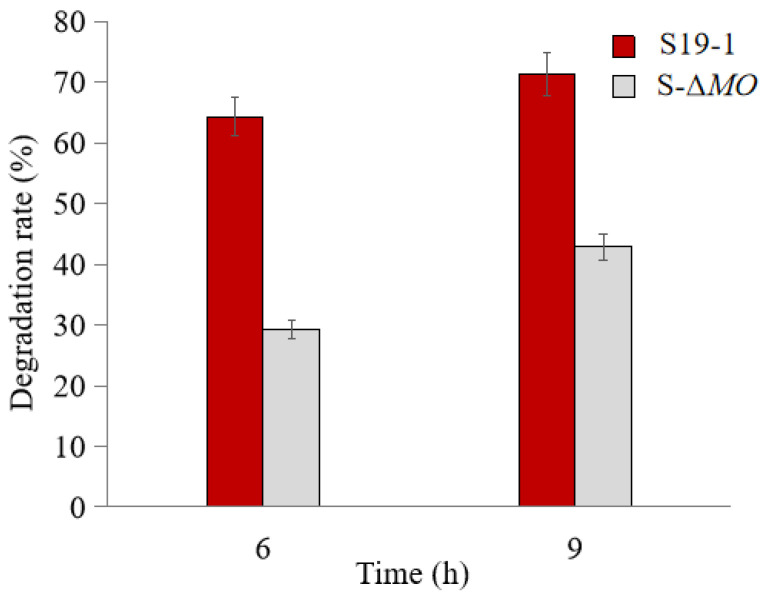
TNT degradation by wild type S19-1 (red) and the S-Δ*MO* (gray) mutant. TNT degradation was assessed after 6 h or 9 h, respectively. Data are presented as mean of *N* = 4, $\bar{x}$ ± SD. Statistical significance is denoted as *p* < 0.05 using SPSS.

HPLC analysis of the cultures after 6 h and 9 h of TNT (1.4 µg·mL^−1^) treatment showed that TNT degradation was significantly less in the BuMO knockout strain (S-Δ*MO)* mutant cultures than in wild-type S19-1 cultures (Figure 4). Exposure to TNT for 6 and 9 h increased the TNT degradation rate of wild-type S19-1 from 64.3% to 71.2% (*p* < 0.05); in contrast, S-Δ*MO* mutant degraded TNT by only 29.3% and 42.9% (*p* < 0.05, Figure 4). This indicates that deficiency of *BuMO* in the S-Δ*MO* mutant significantly hinders its TNT degradation ability; this effect may diminish over time due to potential adaptation by the mutant or the induction of alternative pathways to revamp degradation.

**Figure 5:**
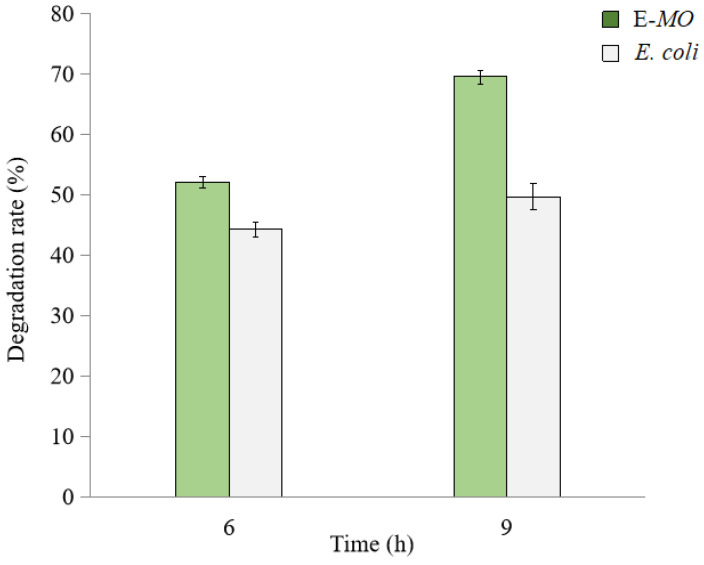
TNT degradation by wild type *E. coli* (gray) and the E-*MO* (green) mutant. TNT degradation was assessed after 6 h or 9 h, respectively. Data are presented as mean of *N* = 4, $\bar{x}$ ± SD. Statistical significance is denoted as *p* < 0.05 using SPSS.

The degradation rate of TNT by *E. coli* was lower than the *BuMO Escherichia coli* expression strain (E-*MO*) mutant, which increased from 45.1% to 50.2% with time without significantly improving the degradation efficiency (Figure 5). In contrast, BuMO expression in the E-*MO* mutant increased TNT degradation from 52.1% to 69.5% by about 1.33-fold, as exposure time increases (*p* < 0.05).

The bacterial transformation of TNT initially utilizes nitroreductases to catalyse the reduction of TNT. Reduced TNT metabolites, such as hydroxyl amino dinitro toluenes (HADNTs) and amino dinitro toluenes (ADNTs), are subsequently susceptible to the action of other oxidative enzymes [34]. Indeed, the broad specificity of MOs to substrates such as toluene and 4-hydroxybenzaldehyde has been widely reported [35]. Although TNT is not a substrate for MO, its catalytic conversion via nitroreductases produces reduced metabolites such as ADNTs and 4-hydroxybenzaldehyde, which may be oxidized by MO [24,25]. Therefore, 4-hydroxybenzaldehyde was selected as the substrate of MO, which plays a vital role in TNT biodegradation.

### 2.4. Gas Chromatography Mass Spectrometry (GC-MS) Analysis

**Figure 6:**
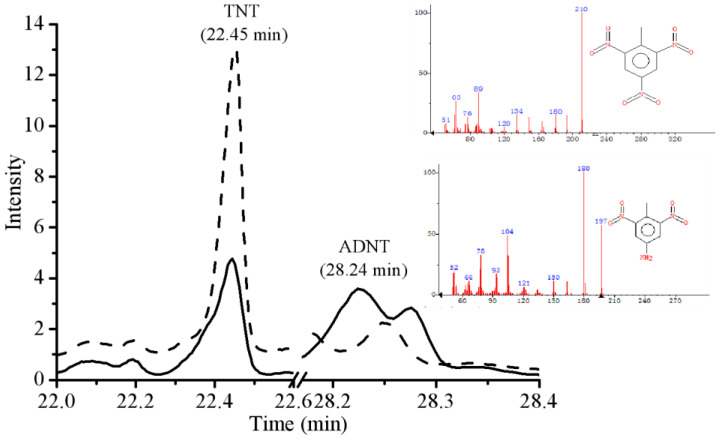
GC-MS analysis of TNT metabolite following degradation. The figure shows peaks of TNT before degradation (short dashes); after 9 h incubation by E-MO (solid lines).

GC-MS analysis of TNT extracts from bacterial cultures prior to incubation showed that the TNT peak appeared at 22.45 min (Figure 6).

GC-MS analysis of E-*MO* (OD_600nm_ = 0.1) after 6 h incubation showed that TNT was degraded to ADNT, and its peak was appeared at 28.24 min (Figure 6). ADNT was the least toxic of TNT and all its dinitrotoluene (DNT) isomers in rats and mice at acute oral doses [36]. Therefore, the toxicity of the solution decreased after bacteria treatment.

To date, the influence of MO in TNT biodegradation by bacteria has been rarely mentioned in existing literature. It is noteworthy that the results presented here are inconsistent with those reported by Serrano-González et al. [24] and Esteve-Núnez et al. [37]. The substrate specificity of MO, as well as its precise role in the process of TNT degradation, are yet to be determined. Indeed, the broad specificity of MOs towards substrates such as toluene [35], methane [38], propane [39], butane [40], and ethene [41] has been widely reported. Hence, the identification of MO substrates from the TNT degradation pathway could facilitate our understanding of its role in the TNT degradation process. In the current study, a knockout of MO in strain S19-1 resulted in a remarkable reduction in TNT degradation in the mutant strain (S-Δ*MO*; Figure 4). This finding also indicates that MO plays a crucial effect in enhancing TNT biodegradation in strain S19-1.

Previous reports have shown that TNT was unaffected by the typical electrophilic attack by oxygenases [42]. Firstly, in the TNT biodegradation pathway, the nitro group of TNT was reduced to hydroxylamine by non-specific NAD(P)H-dependent nitroreductases [13]. Following this, hydroxylamine is reduced to ADNT by nitroreductase. Alternatively, ADNT can also be directly reduced to 2,4,6-triaminotoluene (TAT) by *bifermentans*, *sordelli*, and *sporogenes* [24]. TAT can be catalysed to 2,4,6-trihydroxytoluene using reductase, then forming p-cresol as a result of the reduction of two hydroxide bonds on the aromatic ring of 2,4,6 trihydroxytoluene. p-Cresol is converted to 4-hydroxybenzaldehyde by 4-cresol dehydrogenase and then the methyl group is oxidized by oxygenases to form 4-hydroxybenzoic acid, which can be oxidized to benzoyl-CoA. It then undergoes a range of reactions: to 2,3-epoxybenzoyl-CoA firstly, then to 3,4-dehydroadipyl-CoA-semialdehyde and then to 3, 4-dehydroadipyl-CoA, which is finally converted to acetyl-CoA and succinyl-CoA, and thus to the tricarboxylic acid cycle (TCA) [24,25].

In this research, ADNT was detected using GC-MS analysis after TNT degradation by E-*MO* (following 6-h exposure). This result is consistent with previous reports [24,43]. Indeed, MO has a broad specificity for alkanes and aromatic compounds [28,29,30]. Therefore, MO could participate in the oxidation process of the TNT biodegradation pathway, prior to complete mineralization by the TCA cycle. MO could also be involved in 4-hydroxybenzaldehyde oxidation (a reaction that is known to oxygenases) to form 4-hydroxybenzoate and then oxidized to benzoyl-CoA. Nevertheless, the above proposals still need to be researched and explored in depth in the future.

### 2.5. The Key Enzymes Involved in Biodegradation of TNT in Strain S19-1

**Figure 7:**
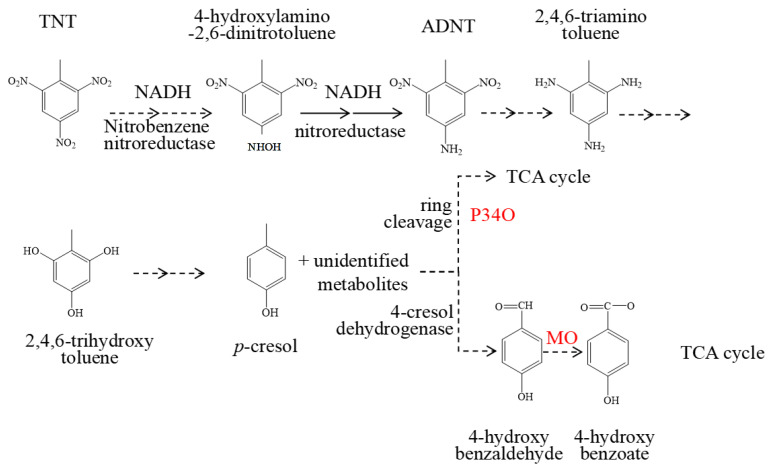
Proposed TNT degradation pathway by P34O and MO.

We previously reported that protocatechuic acid 3, 4-dioxygenase (P34O) is involved in the TNT biodegradation pathway by catalysing the ring cleavage of TNT intermediate metabolites [33]. Herein, we propose that MO may have been involved in the oxidation of 4-hydroxybenzaldehyde to form 4-hydroxybenzoate. Therefore, we speculate that P34O and MO may be involved in different pathways of the TNT biodegradation process (Figure 7). Although P34O acts as dioxygenase and MO acts as monooxygenase, their catalytic sites and degradation pathways are different. Nonetheless, as oxygenases, the catalytic roles of MO and P34O may be similar in the TNT degradation process. To further verify the roles of these key enzymes in TNT degradation, a *P34O/MO* double knockout mutant (S-Δ*M*/Δ*P*) was constructed. As expected, the TNT degradation rate by S-Δ*M*/Δ*P* mutant was significantly less than the wild-type strain S19-1 (approximately a 42% decline) after 6 h exposure (Figure S6). Additionally, the TNT degradation efficiency by the S-Δ*M/*Δ*P* mutant was significantly less than the S-Δ*MO* mutant. Thus, P34O and MO play significant roles in TNT degradation.

In this study, the prokaryotic transcriptome analysis showed that the up-regulated genes, including MO, 1,2-dioxygenase, aldehyde dehydrogenase, oxidoreductase, phosphotransacetylase, alkaline phosphatase, and lysine decarboxylase, may be participants in the TNT biodegradation. This could explain the significantly reduced degradability of the S-Δ*MO* mutant (see Figure 2). It is anticipated that further investigation on the involvement of these genes in the TNT degradation process could also elucidate the TNT biodegradation pathway in strain S19-1.

## 3. Materials and Methods

### 3.1. Culture of Bacteria and Cometabolic Degradation of TNT

The cultivation of bacterial strains was performed as previously reported [33]. Briefly, *Escherichia coli* (DH5α) was incubated in LB medium at 37 °C, and strain S19-1 was incubated in LB medium with 5% NaCl at 27 °C. For cometabolic degradation studies, the overnight culture of strain S19-1 suspension was adjusted to an optical density (OD) of OD_600nm_ = 0.1, with LB medium. Strain S19-1 was exposed to 1.4 µg·mL^−1^ TNT by adding 1 mL LB medium (supplemented with TNT) to 100 µL bacterial suspension (at OD_600nm_ = 0.1). The bacterial suspension was then cultured for 9 h at 180 rpm at 27 °C. LB medium containing 1.4 µg·mL^−1^ TNT was also incubated under the same conditions, in the absence of strain S19-1, to ascertain the degradation rate of TNT by bacteria. Samples extracted prior to incubation (as described in Section 2.2) were used to estimate the initial amount of TNT in cultures before the onset of TNT degradation.

### 3.2. Determination of TNT Concentrations in Bacterial Cultures

TNT was extracted from the cultures after strain S19-1 was incubated in LB medium with TNT by adding 500 µL chloroform to each culture. Samples were oscillated thoroughly and centrifuged at 3000 rpm for 15 min, and then the chloroform phase was collected and centrifuged at 13,000 rpm for 10 min; 300 µL of refined chloroform was then dried at 20 °C in vacuum for 1–2 h. For HPLC analysis, the isolates were reconstituted in 50 µL chromatography grade methanol.

A Shimadzu HPLC system (LC-20AB) with a UV-vis detector (SPD-10A, Shimadzu Corporation, Kyoto, Japan) was selected to identify TNT concentrations in samples at 230 nm. TNT separation was optimized with 60%/40% (*v*/*v*) methanol/water solution as the mobile phase and a flow rate of 700 µL·min^−1^. Samples were analysed at 30 °C with Symmetry C18 column (Waters Corporation, Milford, MA, USA). The injection volume per sample was 5 µL.

TNT degradation efficiency = (1 − P2/P1) × 100%

P1: TNT peak area extracted prior to 9-h culture; P2: TNT peak area extracted after 9-h exposure.

### 3.3. Transcriptomic Analysis of Strain S19-1 under TNT Exposure

#### 3.3.1. Sample Processing

A 100 µL strain S19-1 suspension at OD_600nm_ = 0.1, following overnight cultivation and OD adjustment (see Section 2.1), was cultured in TNT-containing medium (1.4 µg·mL^−1^) at 27 °C for 6 h (T6) and 12 h (T12) at 180 rpm. The control samples were obtained from strain S19-1 cultures incubated for 6 h (C6) and 12 h (C12) in the absence of TNT. RNA was extracted from control and test samples for prokaryotic transcriptomics, using an RNA extraction kit (Sangon Biotech, Shanghai, China) and a Micro BCA protein assay kit (Solarbio, Beijing, China), according to the instructions of manufacturers.

#### 3.3.2. Library Construction, Sequencing, and Bioinformatics Analysis

Construction of RNA-seq libraries and performance of RNA-seq analysis was done according to our previous reports [33]. Data generated from the Illumina platform (San Diego, CA, USA) were used for bioinformatics analysis with I-Sanger Cloud Platform (www.i-sanger.com, accessed on 20 January 2023). The significance of the differences among all genes and transcripts was calculated by *t*-test, and the genes showing statistically significant changes in expression (*p* < 0.05), with log2 fold change > 1, were defined as DEGs. The identified DEGs were analysed by GO and KEGG.

### 3.4. Cloning Vectors and Associated Reagents

pBBR1MCS-2, the vector bearing a kanamycin-resistance gene was provided by Professor Xiong and Professor Maser (Christian-Albrechts-Universität zu Kiel, Kiel, Germany). Ampicillin and kanamycin were obtained from Sigma (Shanghai, China). Bovine alkaline phosphatase and ligase (enzymes used for gene engineering) were obtained from NEB, Sangon Biotech. Standard TNT and ADNT were purchased from the Chinese Academy of Metrology (Beijing, China). Other reagent strains (analytical pure) were purchased from Beijing Chemical Group (Beijing, China).

### 3.5. Isolation and Amplification of Monooxygenase (BuMO) Wild-Type Gene

Following bioinformatic analysis, the full sequence of *BuMO* was isolated and amplified from the strain S19-1 chromosomal DNA via PCR. A point mutation was then generated in the ATG start colon, yielding a shift mutation in the *BuMO* sequence. The primers presented in Table S4 were used—pFM1 and pRM1 (315 bp for *BuMO* in strain S19-1), pFM2 and pRM2 (316 bp for point mutations in Δ*BuMO*), and pFM3 and pRM3 (370 bp for the identification of knockout sequence in recombinants).

### 3.6. Construction of BuMO Expression Vectors

The *BuMO* gene was cloned and expressed in BL21, *E. coli* DH5α, and strain S19-1. The subcloning fragments were chosen in pUcm-T (Sangon, Shanghai China) and pCR2.1-TOPO (Invitrogen, Carlsbad, CA, USA) respectively. Replication of pBBR1MCS-2 was observed in S19-1 and *E. coli* strains. Recombinant DNA approach was adopted from Sambrook et al. [44].

### 3.7. Subcloning of BuMO Full Sequence and ΔBuMO Sequence

As presented in Figure S7, pUcm-T-*BuMO* (pT-*MO*) was produced by cloning the *BuMO* PCR product into pUcm-T. For the knockout plasmid, a point mutation in *BuMO* (Δ*BuMO* 316 bp) was amplified by PCR; this was then inserted into pCR2.1-TOPO to produce pCR2.1-TOPO-Δ*BuMO* (pT-Δ*MO*).

pT-*MO* and pT-Δ*MO* were purified using a SanPrep Column Plasmid Mini-Preps Kit (Ziker, China). Using the CaCl_2_ method, pT-*MO* was transformed into *E. coli* (DH5α) and a *BuMO*-expressing *E. coli* mutant was constructed (E-*MO*, which was resistant to ampicillin). Double transformation of pT-Δ*MO* (with a kanamycin resistance gene) in strain S19-1 was performed. PCR identification of recombinant genes was performed (using primers pFM3 and pRM3, Table S4). Electrophoresis and sequencing were performed as described in Section 2.5 (Figure S8).

The construction of pT-Δ*P34O* was conducted according to previous reports [33]. To ascertain the combined effect of P34O (a key gene involved in TNT-degradation), and MO on TNT degradation in strain S19-1, a *P34O/MO* double knockout mutant (S-Δ*M*/Δ*P*) was constructed using the procedure described above.

### 3.8. Generating the BuMO Knockout Strain (S-ΔMO)

Figures S9A and S9B show the full length and sequence of *BuMO*, respectively. The *BuMO* PCR product was used as a template to isolate and amplify Δ*BuMO* (316 bp) by PCR, using pFM2 and pRM2 (Figure S9B). Δ*BuMO* was subsequently cloned into pCR2.1-TOPO. Resistance to kanamycin enabled isolation of pCR2.1-TOPO-Δ*BuMO* (pT-Δ*MO*; Figure S9C). Figure S9D shows the homologous sequences of *BuMO* and pT-Δ*MO*.

The knockout strain was developed by electrotransfection of strain S19-1 with pT-Δ*MO*. The recombinant clones were confirmed by PCR (using pFM3 and pRM3, Table S4). The amplified length of recombination for the knockout strain (S-Δ*MO*, Figure S8) was 370 bp. Figure S8C shows alignment of the homologous sequence of S-Δ*MO*.

### 3.9. Construction of E-MO (a BuMO-Expressing E. coli Mutant)

The lined pUCm-T and *BuMO* PCR product were cloned to pUCm-T-*BuMO* (pT-*MO*), and then transfected pT-*MO* to *E. coli* DH5α competent cells via the CaCl_2_ method. Ampicillin resistant colonies were isolated.

Using restriction endonucleases (*EcoR* I and *Hind* III), the plasmids from the colonies were single and double-enzyme digested respectively. Restriction-digested pT-*MO* (confirmed by agarose gel electrophoresis) is shown in Figure S10.

### 3.10. Construction of BuMO Expression Vector

Expression vector pET28a and pT-*BuMO* were double digested by *EcoR* I and *Hind* III to create the *BuMO* expression vector (Figure S11). Lined pET28a and *BuMO* full gene were cut from DNA gel and ligated with T4 ligase. The constructed plasmid was then transformed into *E. coli* BL21 competent cells and coated on solid medium with kanamycin for resistance screening. Monoclonal colonies were screened for identification, sequencing, and preservation. The *BuMO* expression vector pET-28a-*BuMO* was obtained (Figure S11).

### 3.11. Bioinformatics Analysis of BuMO

The ExPASy-ProtParam tool (https://web.expasy.org/cgi-bin/protparam/protparam, accessed on 20 January 2023) was used to analyse the physicochemical properties of *BuMO*. The protein secondary and tertiary structures of *BuMO* were predicted in the PSIPRED Protein Analysis Workbench (http://bioinf.cs.ucl.ac.uk/psipred/&uuid=b29e078c-aff9-11ed-9cef-00163e100d53, accessed on 20 January 2023), and SWISS MODLE (https://swissmodel.expasy.org/interactive/8HNH4X/models, accessed on 20 January 2023) separately.

### 3.12. Purification and Analysis of Recombinant BuMO (rBuMO)

To obtain purified rBuMO, pET-28a-*BuMO* was transformed into *E. coli* BL21 and inoculated in LB broth containing 50 mg·L^−1^ kanamycin at 37 °C and 180 rpm. A final concentration of 0.5 mM isopropyl β-D-1-thiogalactopyranoside was added and uniformly mixed into the bacterial suspension to induce rBuMO expression. After incubation for 6 h at 25 °C and 180 rpm, the culture was centrifuged and the precipitate (cell pellet) was resuspended in phosphate buffer solution (PBS, pH 7.4) and then centrifuged again at 13,000 rpm; the cell lysate was located in the supernatant. Based on the properties that rBuMO encoded by pET-28a with six histidine residues and the fact that histidine has a high affinity for nickel-nitrilotriacetic acid, rBuMO was purified using Ni-NTA resin (Qiagen, Dusseldorf, Germany). The concentration of rBuMO was determined by SDS-PAGE and the Bradford assay [45].

4-Hydroxybenzaldehyde, a metabolite of TNT, was selected to be substrate for rBuMO. Usually, monooxygenase is NADH-dependent enzyme, NADH acts as electron donor. Therefore, NADH consumption was used to calculate the rBuMO enzyme activity as the absorbance at 340 nm. The different concentrations of NADH were used to generate a calibration curve.

The reaction solution (300 µL) contained 100 µL rBuMO, final concentration 1 mM NADH, 0.33 mM 4-hydroxybenzaldehyde and then 200 mM Tris-HCl buffer (pH 7.5) diluted to 300 µL [35]. The reaction solution was incubated at 35 °C for different times (5, 10, 30, and 60 min). One unit of enzyme activity was defined as the amount of NADH (µmol) consumed by rBuMO per minute, and rBuMO activity was determined using the formula below:

Specific activity of rBuMO = (C0 − C1) × V/1000 × W × T;

where C0 is the NADH concentration in the blank sample (µM), C1 is the NADH concentration of the experimental sample (µM), V is the reaction volume (mL), W is the amount of enzyme involved in the reaction (mg), and T is the reaction time (min).

### 3.13. Incubation of Strains in TNT-Containing Medium

A 100 μL OD_600nm_ = 0.1 suspension of each of the wild-type strains (S19-1 and *E. coli*) and the two mutants (S-Δ*MO* and E-*MO*) was treated with 1.4 µg·mL^−1^ TNT per 1 mL LB medium and then cultured for 6 and 9 h at 37 °C and 180 rpm. The TNT content of the cultures was analysed using HPLC, as mentioned in Section 2.2. Samples extracted from *E. coli* and E-*MO* cultures, following a 6-h exposure, were assessed via GC-MS.

### 3.14. GC-MS Analysis of TNT Biodegradation Metabolites

A GC (Agilent 7890, capillary column of Agilent DB-5 MS) connected with an MSD (Agilent 5977A) was used to detect the metabolites of TNT in the samples. In splitless mode, the volume of injection was 2 µL. The temperature programming was maintained at 30 °C for 3 min, increasing by 10 °C·min^−1^ to 250 °C, then maintained for 10 min. The injection temperature was 225 °C, and the interface was 250 °C. Helium was selected as the carrier gas and the flow rate was controlled at 1 mL·min^−1^. The samples were analysed in the mode of electron ionization with a mass scan range of 55–550 amu.

## 4. Conclusions

The up-regulation of *MO* in strain S19-1 upon exposure to TNT for 9 h at 180 rpm at 27 °C, and the significant reduction of 1.66-fold (from 71.2% to 42.9%) in degradation efficiency by the S-Δ*MO* mutant, emphasize the relevance of MO in the TNT degradation process. Considering the increase of 1.33-fold (from 52.1% to 69.5%) degradation rate in the E-*MO* mutant, the application of MO to bioremediation systems (such as engineering known TNT-degrading bacteria with enhanced abilities) could boost TNT degradation efforts. Further research on the potential of MO in the degradation of the main TNT metabolites 2- and 4-ADNT and other munition-derived compounds could also prove worthwhile. Therefore, these findings have potential application to bioremediation TNT and its DNT isomers in TNT contaminated sites, such as Northern Germany and the Baltic Sea, where huge amounts of various unused intense explosives were discarded.

## Data Availability

Not applicable.

## Supplementary Materials

The following supporting information can be downloaded at: https://www.mdpi.com/article/10.3390/molecules28041969/s1, Figure S1: GO enrichment analysis in 6 h degradation groups; Figure S2: GO enrichment analysis in 12 h degradation groups; Figure S3: KEGG enrichment analysis of DEGs in 6 h degradation groups; Figure S4: KEGG enrichment analysis of DEGs in 12 h degradation groups; Figure S5: Spatiotemporal series analysis; Figure S6: TNT degradation by wild type S19-1 and the S-Δ*M*/Δ*P* mutant for 6 h; Figure S7: Schematic illustration of knockout and expression constructs; Figure S8: Identification of recombinant strain S-Δ*MO*; Figure S9: Cloning of the full sequence of *BuMO* and Δ*BuMO* genes; Figure S10: Agarose gel of restriction-digested pUCm-T-*BuMO*; Figure S11: Construction of *BuMO* expressing-vector with pET28a and pUCm-T-*BuMO*. Table S1: Statistics of filtered reads quality; Table S2: Up-regulation of differentially expressed genes top 10 in TNT degradation group (6 h/12 h); Table S3: Analysis of monooxygenase in TNT degradation groups; Table S4: Primers used in this study.

## Sample Availability

Samples of *Buttiauxella* sp. S19-1 and pBBR1MCS-2 are available from the authors.

## Abbreviations

TNT, 2-amino-4,6-dinitrotoluene; WAO, wet air oxidation process; ADNT, 4-amino-2,6-dinitrotoluene; MO, monooxygenases; PAHs, polycyclic aromatic hydrocarbons; OD, optical density; HPLC, high-performance liquid chromatography; DEG, differentially expressed genes; GO, gene ontology; KEGG, Kyoto Encyclopaedia of Genes and Genomes; BuMO, isolation and amplification of monooxygenase; PCR, polymerase chain reactions; Δ*BuMO*, a point mutation in *BuMO*; pT-*MO*, pUcm-T-*BuMO*; pT-Δ*MO*, pCR2.1-TOPO-ΔB*uMO*; E-*MO*, *BuMO Escherichia coli* expression strain; P34O*,* protocatechuic acid 3, 4-dioxygenase; S-Δ*M*/Δ*P*, *P34O/MO* double knockout S19-1 mutant; S-Δ*MO*, absence of *BuMO* gene in S19-1 mutant; pT-*BuMO*, pET-28a-*BuMO*; rBuMO, purified recombinant BuMO; PBS, phosphate buffer solution; GC-MS, gas chromatography mass spectrometry; PTS, phosphotransferase; gap, glyceraldehyde 3-phosphate dehydrogenase; leuO, LysR family transcriptional regulator; wcaD, putative colanic acid polymerase; bioC, malonyl-CoA O-methyltransferase; uca, urea carboxylase; urtB, urea transport system permease protein; tehA, tellurite resistance protein; HADNTs, hydroxyl amino dinitro toluenes; ADNTs, amino dinitro toluenes; DNT, dinitrotoluene; TAT, 2,4,6-triaminotoluene; TCA, tricarboxylic acid cycle.
