# Supplementary material for: Critical Role of Monooxygenase in Biodegradation of 2,4,6-Trinitrotoluene by Buttiauxella sp. S19-1
Source: Molecules. 2023 Feb 19;28(4):1969. doi: 10.3390/molecules28041969 (PMC9958683; doi:10.3390/molecules28041969)
Supplement: Supplementary file 1 [file molecules-28-01969-s001.zip › molecules-2204347-supplementary.pdf]

Supplementary Materials

Critical role of monooxygenase in biodegradation of 2,4,6-trinitrotoluene by *Buttiauxella* sp. S19-1

Miao Xu[#], Lei He, Ping Su[1#], Ming Wu, Xiyan Cui, Dong Liu, Amma G. Adomako-Bonsu, Min Geng, Guangming Xiong, Liquan Guo* and Edmund Maser*

Figures

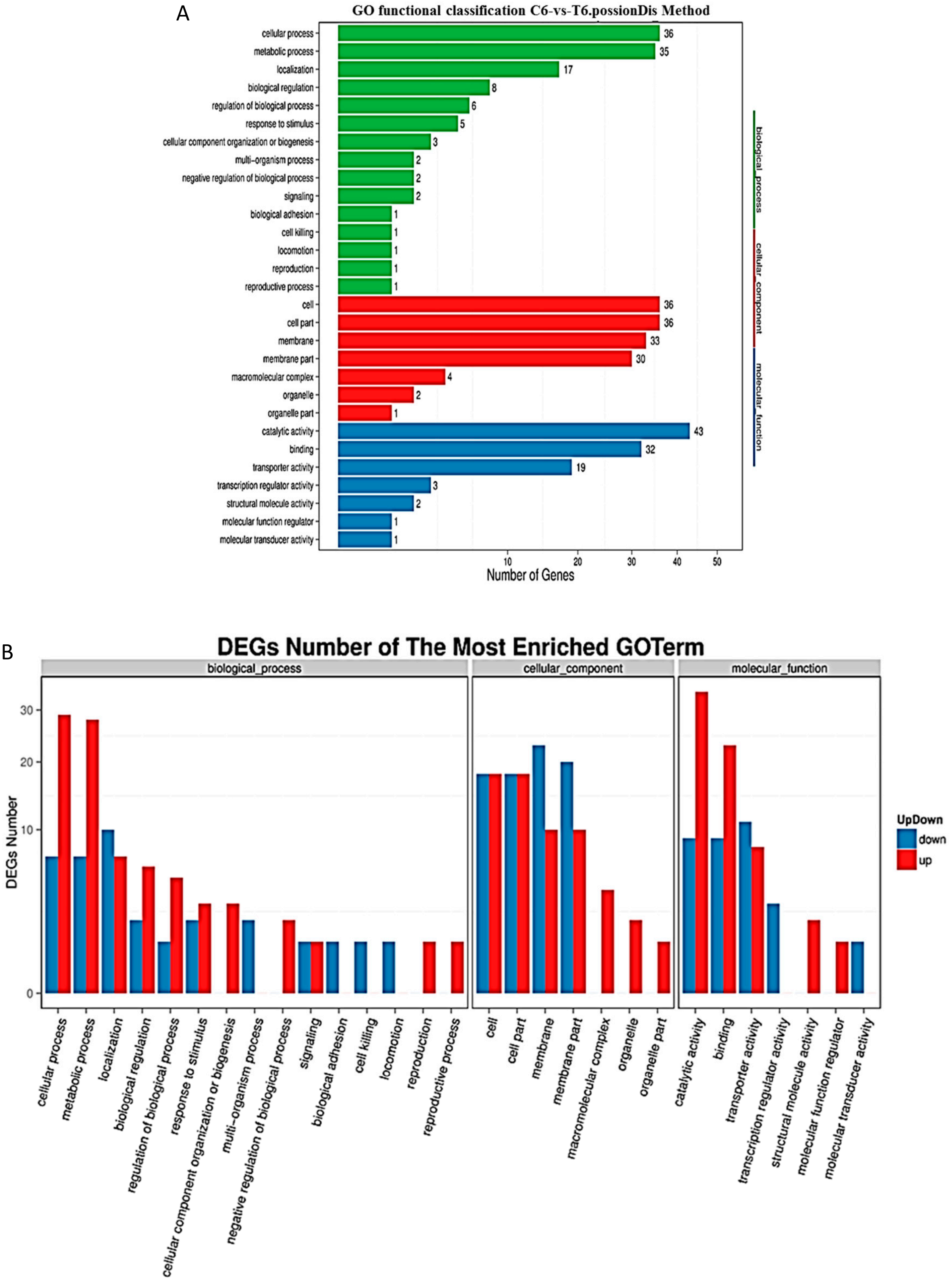

Figure S1. GO enrichment analysis in 6 h degradation groups.

A- Distribution of DEGs for GO enrichment analysis. **B**- GO enrichment analysis of DEGs

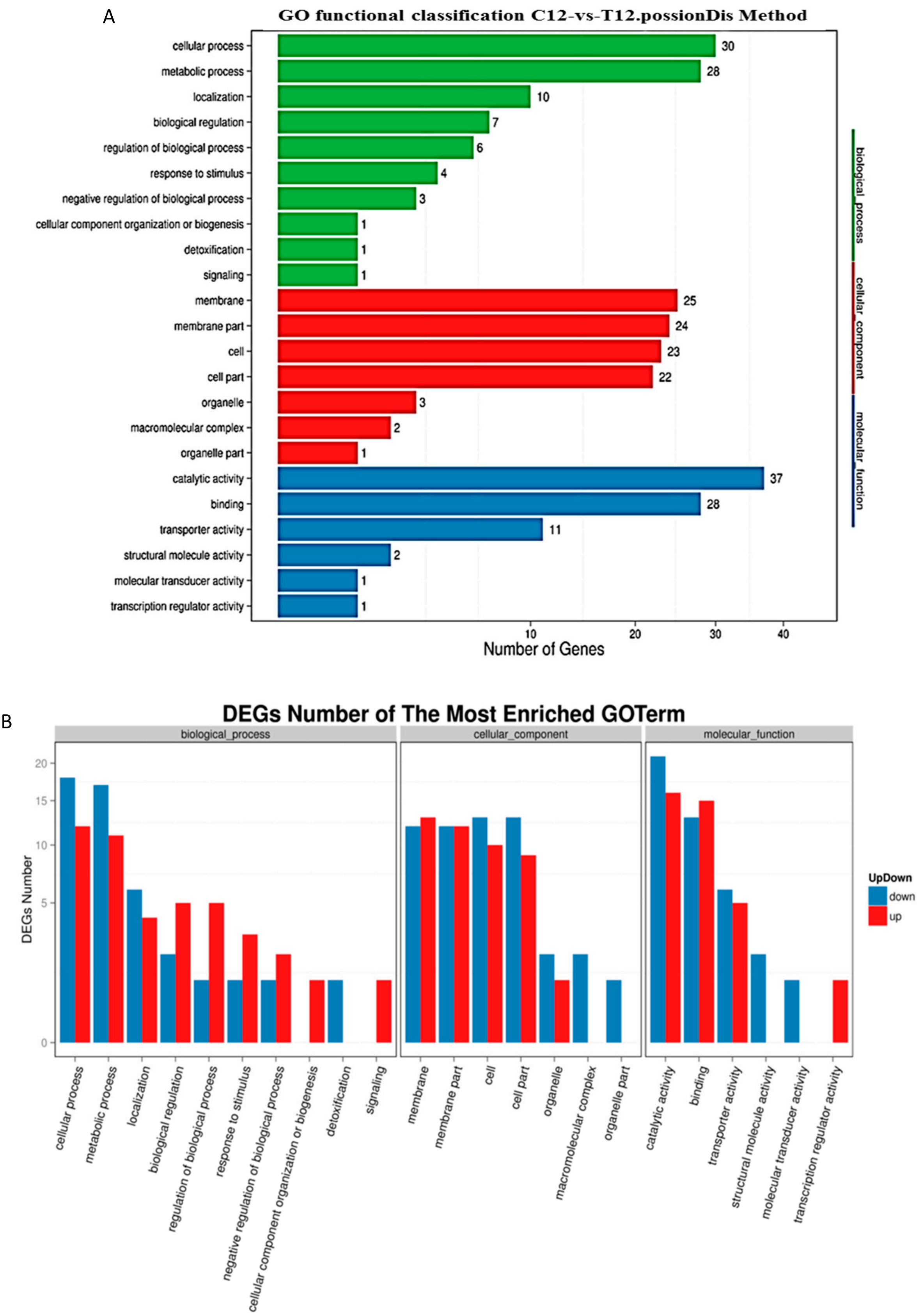

Figure S2. GO enrichment analysis in 12 h degradation groups.

A- Distribution of differential genes for GO enrichment analysis. **B**- GO enrichment analysis of DEGs.

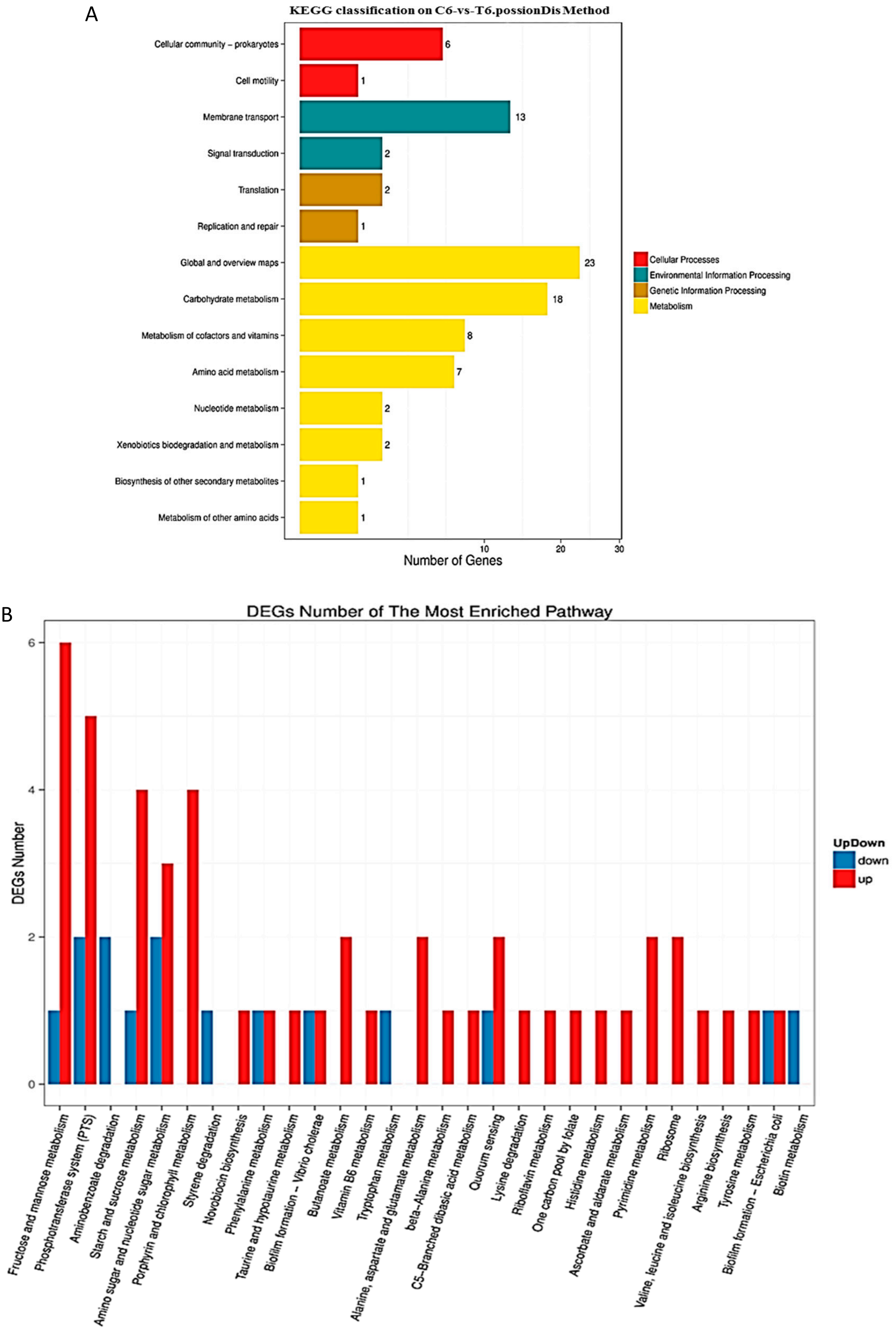

Figure S3. KEGG enrichment analysis of DEGs in 6 h degradation groups.
A-KEGG enrichment analysis of DEGs. **B**-Enrichment analysis of DEGs

A

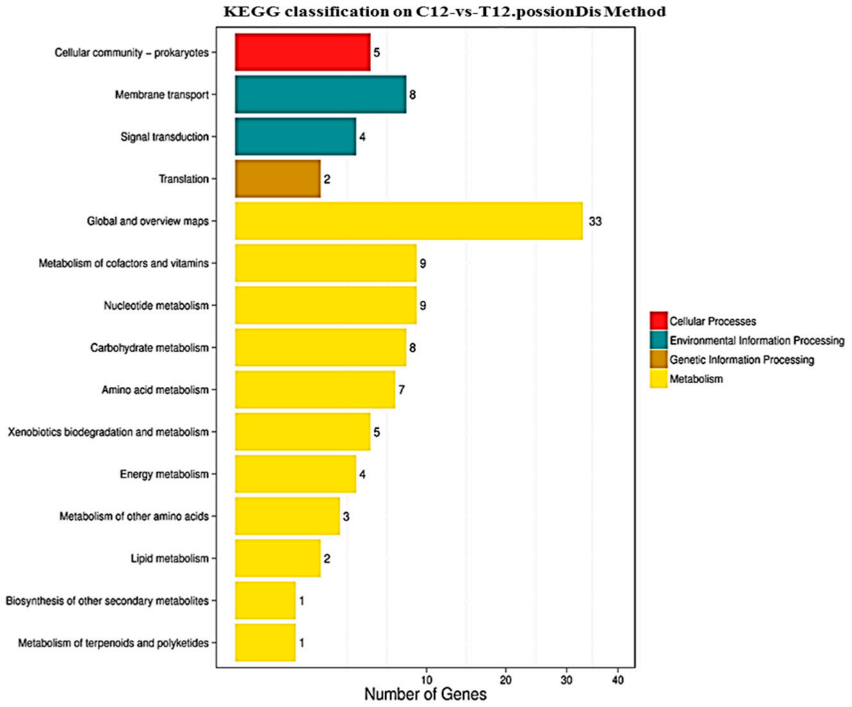

B

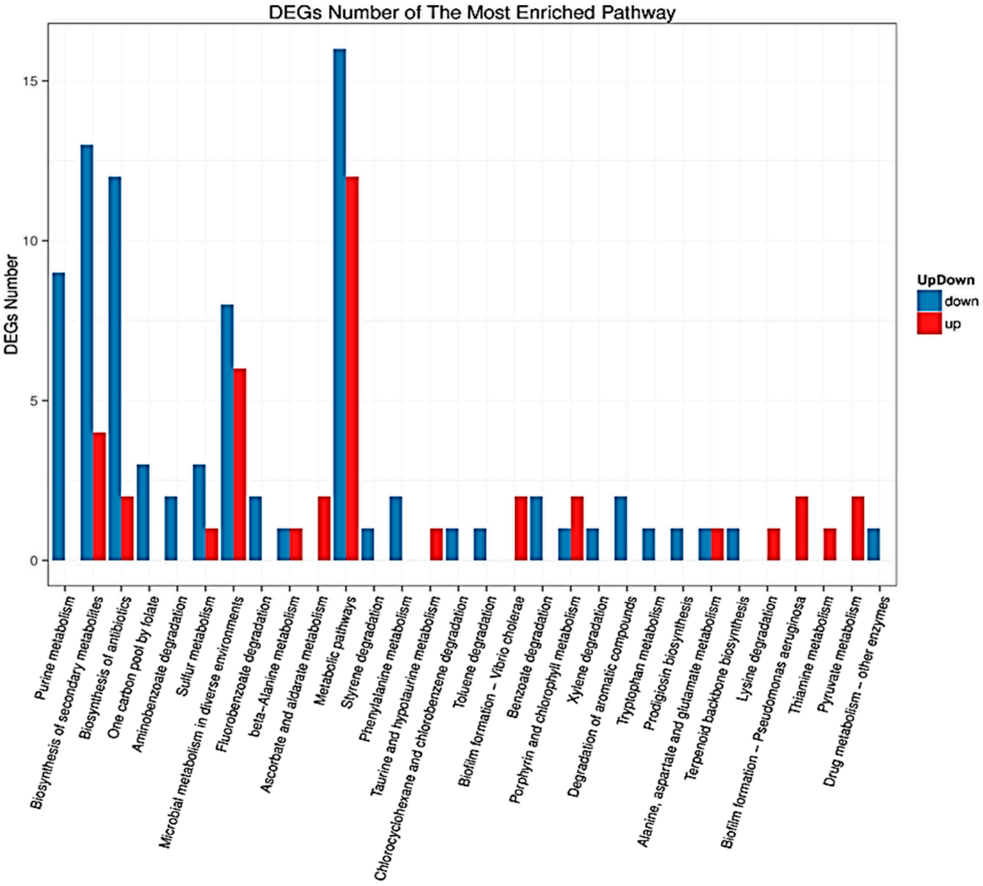

Figure S4. KEGG enrichment analysis of DEGs in 12 h degradation groups. **A**-KEGG enrichment analysis of DEGs. **B**-Enrichment analysis of DEGs.

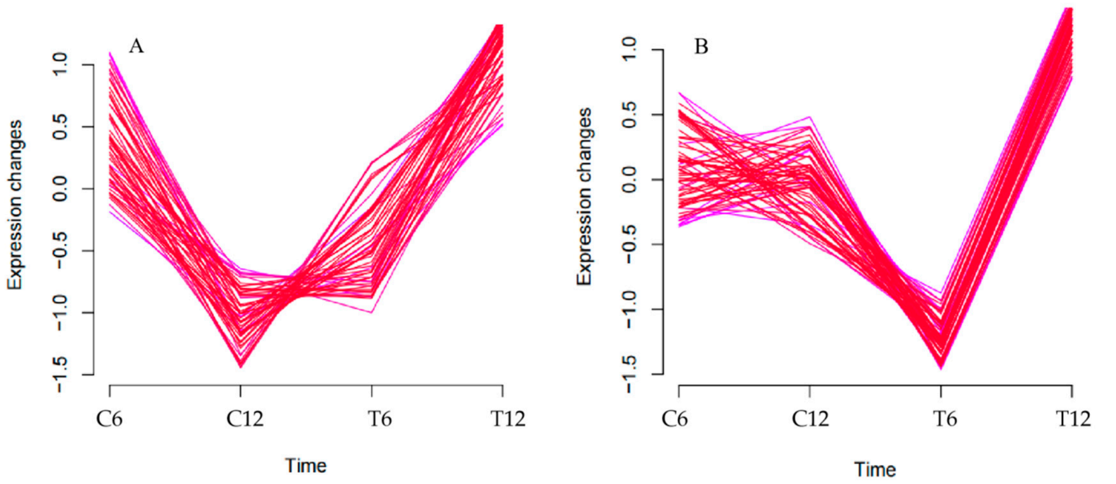

Figure S5. Spatiotemporal series analysis.

A-Expression changes at 6 h and 12 h. **B**-Expression changes between control groups (C) and TNT groups (T).

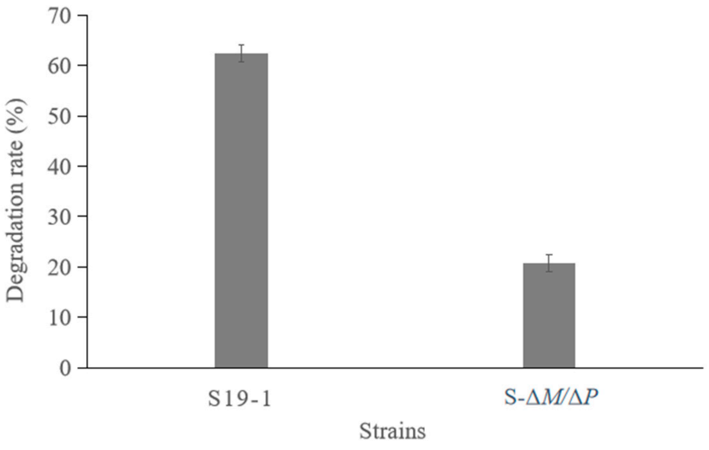

Figure S6. TNT degradation by wild type S19-1 and the S-Δ*M*/Δ*P* mutant for 6 h.

Data are presented as mean of N = 4, $\bar{x}$ ± SD. Statistical significance is denoted as $p < 0.05$ using SPSS.

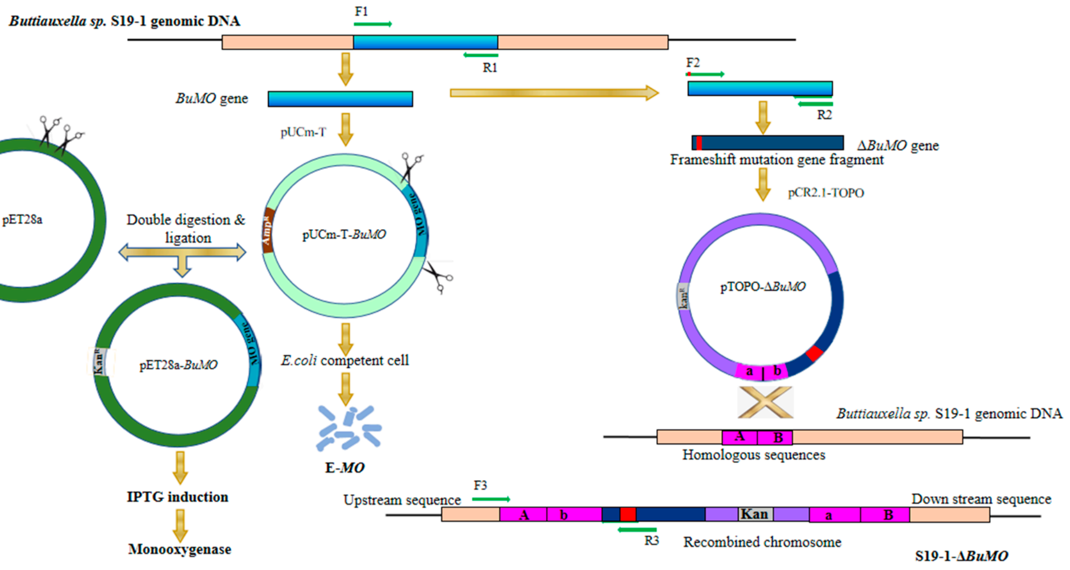

Figure. S7 Schematic illustration of knockout and expression constructs.

Expression and knockout fragment are represented by blue and dark blue respectively; the inserted base is represented by red; genomic DNA and homologous sequences in strain S19-1 are represented by orange and pink; plasmid DNA is represented by light blue, green and purple respectively; resistance genes represented by brown and light gray.

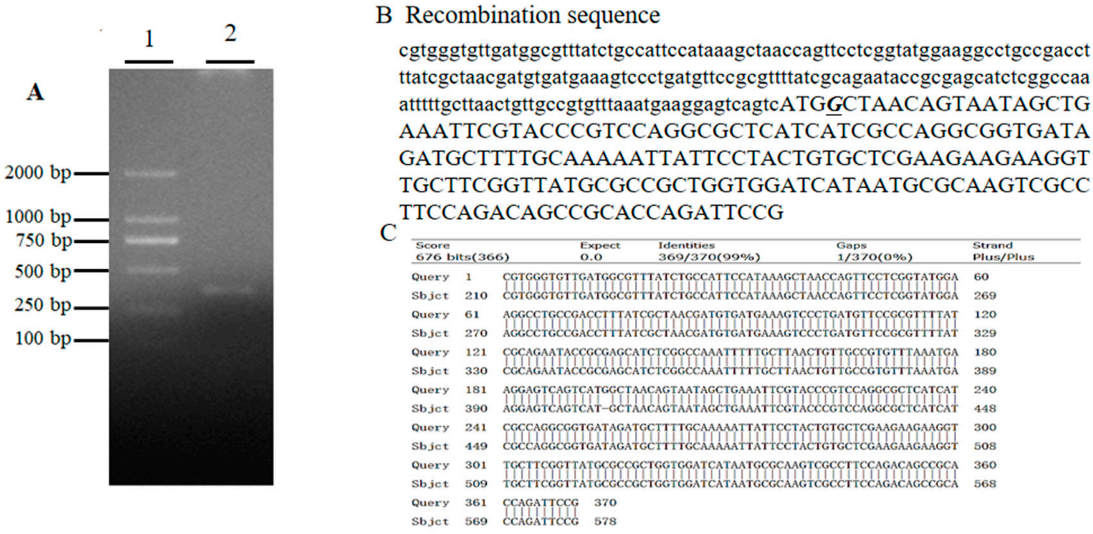

Figure S8. Identification of recombinant strain S-Δ*MO*.

A-gel showing size marker (1) and PCR product of recombination (2). **B**-sequence of recombination; genes labelled with lowercase letters are located on *Buttiauxella* sp. S19-1 genome before *BuMO* sequence. Genes labelled with capitalized letters are located on the plasmid. Insert bases are labelled, underlined and displayed in bold. **C**-homologous sequence alignment between sequences of recombination and *Buttiauxella* sp. S19-1 genome.

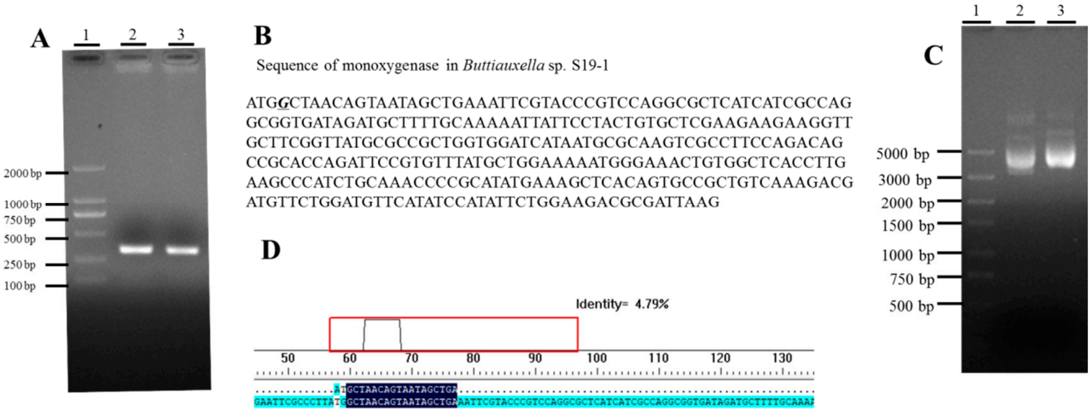

Figure S9. Cloning of the full sequence of *BuMO* and Δ*BuMO* genes.

A-gel showing size marker (1), PCR of *BuMO* (315 bp, 2) and Δ*BuMO* (316 bp, 3); **B**-Sequence of monoxygenase in *Buttiauxella* sp S19-1; **C**-gel showing size marker (1), pCR2.1-TOPO (3931 bp, 2) and pTOPO-Δ*BuMO* (pT-*MO* 4250 bp, 3); **D**-blast of sequences between *BuMO* and pT-Δ*MO*.

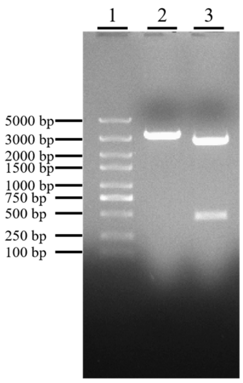

Figure S10. Agarose gel of restriction-digested pUCm-T-*BuMO*.

Gel showing single/double digested product of pUCm-T-*BuMO* by *EcoR* I and *Hind* III (double digested sites are located on both sides of the multiple cloning site of pUCm-T) respectively. The size marker is labelled appropriately (1); the size of the product of pUCm-T-*BuMO* (2) is approximately 3088 bp and the double digested product of pUCm-T-*BuMO* (3) is approximately 2635 bp and 453 bp.

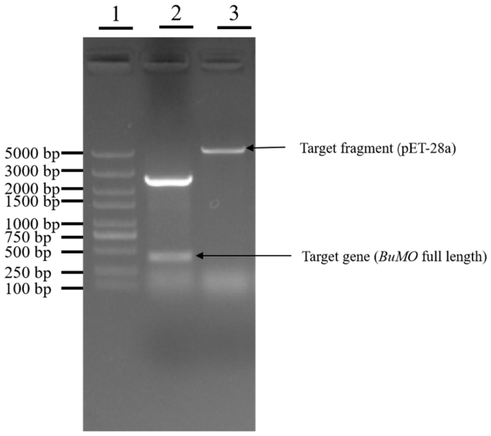

Figure S11. Construction of *BuMO* expressing-vector with pET28a and pUCm-T-*BuMO*.

Tables

Table S1. Statistics of filtered reads quality.

Samples	Total Raw Reads (Mb)	Total Clean Reads (Mb)	Total Clean bases (Gb)	Clean Reads Q20 (%)	Clean Reads Q30 (%)	N Reads (%)	Low Quality Reads (%)	Adapter Reads (%)	Other Reads (%)	GC content (%)	Clean Read Ratio (%)
C12	16.33	15.02	2.25	99.15	97.34	0.01	2.14	5.83	0.00	51.88	92.01
C6	16.33	15.31	2.30	98.98	96.88	0.01	3.05	3.17	0.00	52.94	93.78
T12	16.33	15.12	2.27	99.09	97.19	0.01	2.12	5.27	0.00	52.20	92.60
T6	16.33	14.94	2.24	99.11	97.22	0.01	2.35	6.15	0.00	52.66	91.49

Q20: The quality value is greater than 20, indicating that the base error rate is 1%.

Q30: The quality value is greater than 30, indicating that the base error rate is 0.1%.

Ps: Strain S19-1 was cultured in TNT-containing medium for 6 h (T6) and 12 h (T12). The control samples were obtained from strain S19-1 cultures incubated for 6 h (C6) and 12 h (C12) in the absence of TNT.

Table S2. Up-regulation of differentially expressed genes top10 in TNT degradation group (6h/12h).

Gene ID	Sets	Length	Log2 FC	Significance	GO	KEGG
mar	6h	210	8.501173724	P<0.01	-	K13630
hycH	6h	411	7.348728154	P<0.01	GO:0008047	K15834
sgrT	6h	177	6.661956343	P<0.01	GO:0046325	-
D8682_RS02970	6h	228	3.414039716	P<0.01	-	-
D8682_RS04610	6h	240	3.278254252	P<0.01	-	-
D8682_RS04560	6h	243	3.131582799	P<0.01	-	-
D8682_RS10315	6h	366	2.944756058	P<0.01	GO:0003677	K02237
D8682_RS19250	6h	1179	2.928001676	P<0.01	GO:0015542	K03291
D8682_RS22070	6h	1242	2.896608819	P<0.01	GO:0015542	-
D8682_RS24265	6h	282	2.696634137	P<0.01	GO:0015087	K02009
D8682_RS26110	12h	183	10.7968508	P<0.01	-	-
D8682_RS20940	12h	291	9.121533517	P<0.01	GO:0003677	K07724
D8682_RS06365	12h	495	7.988684687	P<0.01	-	K11903
D8682_RS19245	12h	177	5.697772294	P<0.01	GO:0046325	-
D8682_RS01190	12h	999	5.087462841	P<0.01	GO:0016021	K14347
D8682_RS26465	12h	999	5.087462841	P<0.01	GO:0016021	K14347
D8682_RS11885	12h	399	3.662965013	P<0.01	-	-
D8682_RS02235	12h	603	3.074109214	P<0.01	-	-
D8682_RS16090	12h	696	2.870716983	P<0.01	GO:0016021	K10921
D8682_RS11975	12h	318	2.525478068	P<0.01	GO:0004497	K04025

Table S3. Analysis of monooxygenase in TNT degradation groups.

geneID	geneLength	CK12-FPKM	TNT12-FPKM	log2FoldChange (TNT12/CK12)	Up-Down-Regulation (TNT12/CK12)	FDR	P-value	type	GO Function	Blast nr
D8682_RS11975	318	150.64	308.64	2.52548	Up	5.28E-15	3.23E-16	known_mRNA	GO:0004497//monooxygenase activity	WP_064540086.1/9.1e-53/1

Table S4. Primers used in this study.

Primer name	Sequence
pFM1	5′ ATGCTAACAGTAATA-3′
pRM1	5′-CTTAATCGCGTCTTC-3′
PFM2	5′-ATGGCTAACAGTAATA-3′
pRM2	5′-CTTAATCGCGTCTTC-3′
PFM3	5′-CGTGGGTGTTGATGGCGTTT-3′
pRM3	5′-CGGAATCTGGTGCGGCTGT-3′
